# Multiple meningiomatosis

**DOI:** 10.11604/pamj.2021.40.59.30098

**Published:** 2021-09-24

**Authors:** Inas El Kacemi, Gazzaz Miloudi

**Affiliations:** 1 Service de Neurochirurgie, Hôpital Militaire d´Instruction Mohammed V de Rabat, Rabat, Maroc,; 2 Faculté de Médecine et de Pharmacie, Université Mohammed V de Rabat, Rabat, Maroc

**Keywords:** Multiple meningiomas, meningiomatosis, magnetic resonance imaging (MRI)

## Image in medicine

Multiple meningiomas or meningiomatosis are defined by the presence of at least 2 lesions that appear simultaneously or not, at different intracranial locations. Early classification of Cushing and Eisenhardt claimed that a diagnosis of multiple meningioma can only be made if the patient does not suffer neurofibromatosis type I (NF1 is associated with neurofibromin, ras pathway). Majority of multiple meningiomas associate with neurofibromatosis type-II, multiple meningiomatosis refers to the association of at least 2 tumors in two different sites, in a patient who has no evidence of neurofibromatosis. The incidence of this condition varies between series between 1 and 3%, reaching a frequency of 8% with the onset of magnetic resonance imaging (MRI). This entity combines benign tumors of a different histological nature in 30% of cases. A 39-year-old woman patient, with no significant pathological history, who consulted for heaviness in both lower limbs having progressed for 6 months with paresthesias without genitosphincteric disorders, the examination found a spastic paraparesis rated at 3/5. The patient underwent a medullary MRI which revealed 4 lesions, the radiological semiology of which was suggestive of spinal meningiomas, the most compressive projecting in relation to T2. A cerebral computed tomography (CT) was systematically performed and objectified two intra cranial meningiomas, which until then were asymptomatic. Although its incidence is only 1 to 3%, the discovery of multiple meningiomatosis justifies morphological exploration (MRI) of the entire neurax, in which case any symptomatic location should suggest surgical management.

**Figure 1:**
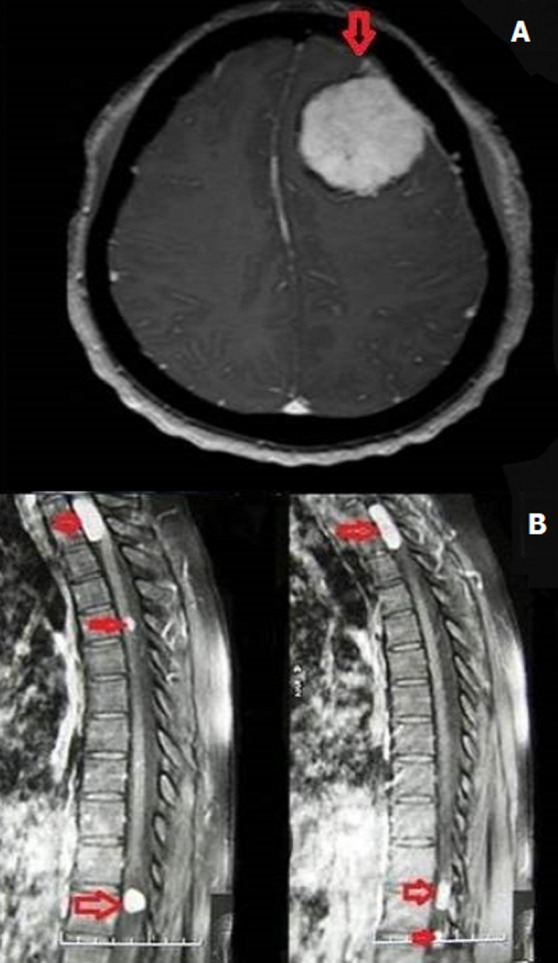
(A,B) brain MRI with contrast axial section showing frontal convexity meningioma, spine MRI on sagittal sections on T1 injection showing 4 lesions, the most compressive meningiomas are projected in T2 whose major axis is 28 mm

